# Identification of a novel ubiquitination related gene signature for patients with breast cancer

**DOI:** 10.1097/MD.0000000000030598

**Published:** 2022-09-16

**Authors:** Yuan Zheng, Wenliang Lu, Bo Chen, Kankan Zhao

**Affiliations:** a Department of Thyroid and Breast Surgery, Maternal and Child Health Hospital of Hubei Province, Tongji Medical College, Huazhong University of Science and Technology, Wuhan City, P.R. China.

**Keywords:** bioinformatics analysis, breast cancer, prognosis signature, ubiquitination

## Abstract

Ubiquitination related genes (URGs) are important biomarkers and therapeutic targets in cancer. However, URG prognostic prediction models have not been established in breast cancer (BC) before. Our study aimed to identify URGs to serve as potential prognostic indicators in patients with BC.The URGs were downloaded from the ubiquitin and ubiquitin-like conjugation database. GSE42568 and The Cancer Genome Atlas were exploited to screen differentially expressed URGs in BC. The univariate Cox proportional hazards regression analysis, least absolute shrinkage and selection operator analysis, and multivariate Cox proportional hazards regression analysis were employed to construct multi-URG signature in the training set (GSE42568). Kaplan–Meier curve and log-rank method analysis, and ROC curve were applied to validate the predictive ability of the multi-URG signature in BC. Next, we validated the signature in test set (GSE20685). Finally, we performed GSEA analysis to explore the mechanism.We developed a 4-URG (CDC20, PCGF2, UBE2S, and SOCS2) signature with good performance for patients with BC. According to this signature, BC patients can be classified into a high-risk and a low-risk group with significantly different overall survival. The predictive ability of this signature was favorable in the test set. Univariate and multivariate Cox regression analysis showed that the 4-URG signature was independent risk factor for BC patients. GSEA analysis showed that the 4-URG signature may related to the function of DNA replication, DNA repair, and cell cycle.Our study developed a novel 4-URG signature as a potential indicator for BC.

## 1. Introduction

Breast cancer (BC) ranks first in terms of incidence among all cancers according to statistics from International Agency for Research on Cancer.^[1]^ Although novel therapies including targeted therapy and immune therapy are implemented in clinical practice and clinical trial design, clinical outcomes for BC remain unsatisfactory. There are also promising treatments of medical plants with less toxicity.^[2]^ The anticancer mechanism of medical plant is versatile such as stimulation of death, promotion of cell cycle arrest, inhibition of cell invasion and migration and so on, but it needs time and large clinical trial for these medical plants be widely used. With advances in cancer biology, it is possible to develope compounds anchoring small molecules to macromolecules that act with specific mechanism such as DNA activation, or tubulin polymerization.^[3]^ Nevertheless, these compounds are lacked. Tailored treatment based on molecules which are involved in the growth, progression and metastasis of BC is meaningful for improving the outcomes. However, these molecules are limited. Thus, it is of vital importance to identify novel molecules contribute to risk stratification and clinical-decision making.

Ubiquitination is one of the most common and important posttranslational modifications. Ubiquitin preoteasome system is a highly-specific, adenosine triphosphate-dependent pathway regulating specific proteins degradation in eucaryote. Ubiquitination is a reversible process, which was mediated by 3 types of enzymes, E1 ubiquitin activating enzyme, E2 ubiquitin conjugating enzyme, and E3 ubiquitin ligase.^[4]^ E1 activates ubiquitin and transfer it to its activate site Cys in the adenosine triphosphate-dependent manner. E2 transports ubiquitin to E2 itself by binding E1. E3 recognizes substrate proteins and catalyzes ubiquitin transfer from E2 to the substrate. Proteins labeled with ubiquitin are finally taken to the proteasome for degradation. There are other ubiquitin-like modifications, including SUMOylation, Pupylation, and ISGlation.^[5]^ The process can be reversed by deubiquitinating enzymes to cleave ubiquitin and ubiquitin-like from the substrate. In addition, the ubiquitin also has many nondegradative functions.^[6]^ As reported by other studies,^[7,8]^ ubiquitination plays important roles in many cell signaling pathways and biological processes, such as protein activation and transactivation, DNA replication and repair, cell cycle, chromatin dynamics, transcription signaling transduction, autophagy, and immune response, suggesting them as important biomarkers and therapeutic targets. However, ubiquitination related gene (URG) prognostic prediction models have not been established in BC before.

In the present study, we exploited gene expression Omnibus (GEO) database and The Cancer Genome Atlas (TCGA) to screen differentially expressed prognostic URGs. Based on these prognostic URGs, we identified a 4-URG signature with good performance for patients with BC. Our analysis suggests URGs play important roles in BC and were potential prognostic biomarkers.

## 2. Materials and Methods

### 2.1. Data collection and processing

We applied GEO database^[9]^ (http://www.ncbi.nlm.nih.gov/geo/) (GSE42568 and GSE20685) to acquire the gene expression quantification data and corresponding clinic data of patients with BC. We applied GEO2R^[10]^ to identify differentially expressed genes in GSE42568 dataset^[11]^ (104 BC samples and 17 normal samples) according to the threshold |log2 fold change (log2FC)| > 1 and adjust *P* < .05. RNA-seq data of patients with BC were retrieved from the TCGA (http://portal.gdc.cancer.gov) (112 adjacent normal samples and 1089 primary solid tumor samples). We applied limma package^[12]^ to identify differentially expressed genes based on the same threshold. The volcano plots were plotted to visualize differentially expressed genes in GSE42568 dataset and TCGA. The URGs were downloaded from the ubiquitin and ubiquitin-like conjugation database^[13]^ (http://uucd.biocuckoo.org). We merged the URGs and differentially expressed genes in GSE42568 dataset and TCGA to acquire differentially expressed URGs expression. 327 BC patients in GSE20685^[14]^ with gene expression data and baseline data were included as validation set. The univariate Cox proportional hazards regression analysis was used to explore the association of differentially expressed URGs with BC patients’ overall survival (OS) and OS time in the training set (GSE42568). Those URGs with *P* value < .05 were considered prognostic URGs.

### 2.2. Construction, evaluation, and validation of URG signature in BC

The prognostic URGs were entered into the least absolute shrinkage and selection operator (LASSO) method analysis to select the best candidates in the training set (GSE42568) by using glmnet package^[15]^ in R. Then, we conducted multivariate Cox proportional hazards regression analysis to develop the multi-URG signature using the candidates resulting from LASSO analysis. Based on the corresponding coefficients and expression of selected genes, the URG signature was constructed as follows: risk score = (β_1_*Gene_1_Exp + β_2_* Gene_2_Exp + β_3_* Gene_3_Exp + ··· + β_n_* Gene_n_Exp). In this formula, β represents the coefficients in the multivariate Cox regression analysis. Next, we calculated the risk score for each BC patients and classified BC patients into a high-risk and low-risk group according to the median risk score. The Kaplan–Meier curve and log-rank method were performed to evaluate the OS difference between the high-risk and low-risk group. We used time-dependent receiver operating characteristic (ROC) curve to assessed the sensitivity and specificity of the URG signature by calculating the area under the curve (AUC).^[16,17]^ We applied the multi-URG signature in the test set (GSE20685) to verify the reliability.

### 2.3. Independence of UGR signature in BC and correlation of URG with clinicopathological factors

To verify the independence of the prognostic value of the multi-URG signature based risk score and clinicopathological factors (including age, grade, tumor size, lymph node status, and metastasis status), univariate and multivariate Cox regression analyses were performed to explore their associations with OS of BC patients. Factors with *P* value <.05 in the multivariate Cox regression analysis were considered independent factors. We also evaluated the association of URGs in the multi-URG signature with clinicopathological factors.

### 2.4. Functional enrichment analysis

In order to reveal the heterogeneity between high-risk group and low-risk group patients, we performed gene set enrichment analysis (GSEA).^[18]^ Gene Ontology and the Kyoto Encyclopedia of Genes and Genomes pathway^[19]^ gene sets were selected as the reference gene sets. The results of GSEA were visualized using enrichplot package^[20]^ in R language.

### 2.5. Statistical methods

All statistical analyses were performed by R software (version 4.1.3). Wilcoxon rank-sum test was used to compare the difference between the 2 groups, Kruskal–Wallis test was performed to compare the difference of 3 or more groups. Kaplan–Meier curve and log-rank method were performed to evaluate the OS difference between groups. The ROC curves were plotted to assessed the sensitivity and specificity of the URG signature. A 2-tailed *P* value <.05 was considered statistically significant.

## 3. Results

### 3.1. Identification of prognostic URGs in BC

**Figure 1.**
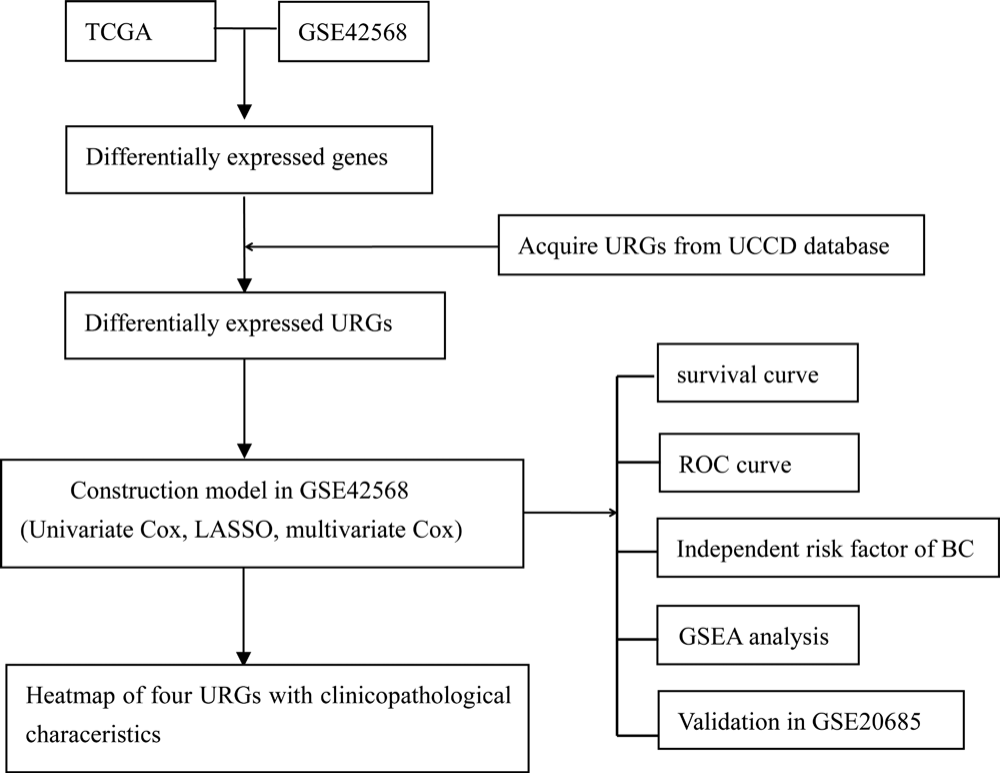
The workflow of the present study. BC = breast cancer, GSEA = gene set enrichment analysis, ROC = receiver operating characteristic, TCGA = The Cancer Genome Atlas, URGs = ubiquitination related genes, UUCD = ubiquitin and ubiquitin-like conjugation database.

**Figure 2.**
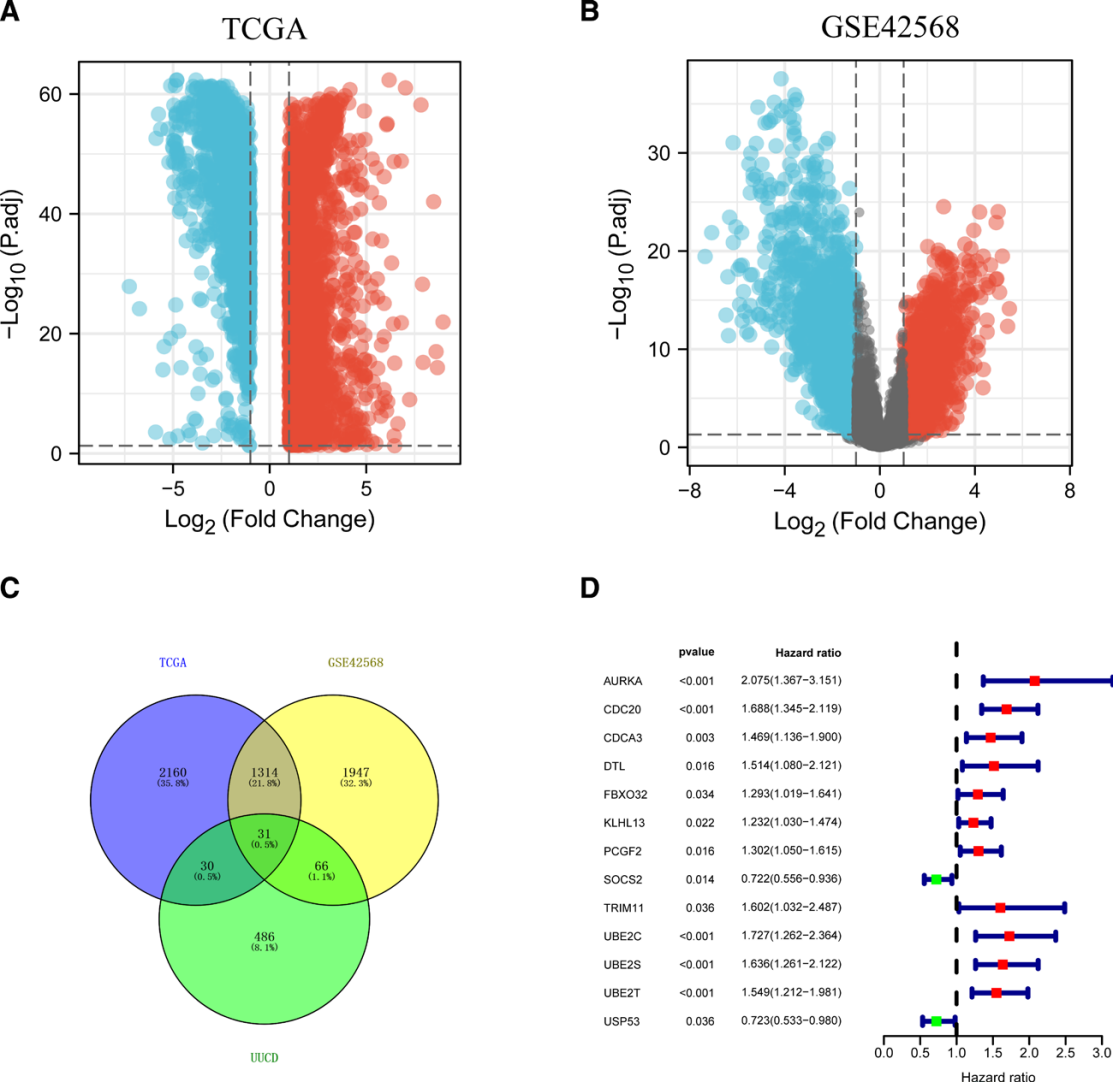
Identification of differentially expressed URGs in breast cancer. (A) Volcano plot of differentially expressed genes in TCGA. (B) Volcano plot of differentially expressed genes in GSE42568 dataset. (C) Venn plot of URGs and differentially expressed genes. (D) Univariate Cox hazards regression analysis of URGs with OS of breast cancer patients. OS = overall survival, TCGA = The Cancer Genome Atlas, URG = ubiquitination related gene.

The workflow of the present study is presented in Figure 1. As Figure 2 showed, a total of 61 differentially expressed URGs in TCGA (Fig. 2A), and 97 differentially expressed URGs in GSE42568 (Fig. 2B) were identified. As a result, we acquired 31 common differentially expressed URGs in both datasets (Fig. 2C). These URGs were further assessed for their association with the survival of BC in the training group (GSE42568). 13 URGs were found to be significantly associated with OS of BC by univariate Cox proportional hazards regression analysis (Fig. 2D). Among these 13 URGs, SOCS2 and USP53 were found to be protective factors of BC patients (hazard ratio < 1), and the remaining 11 URGs were risk factors of BC patients (hazard ratio > 1).

### 3.2. Construction, evaluation, and validation of URG signature in BC

**Figure 3.**
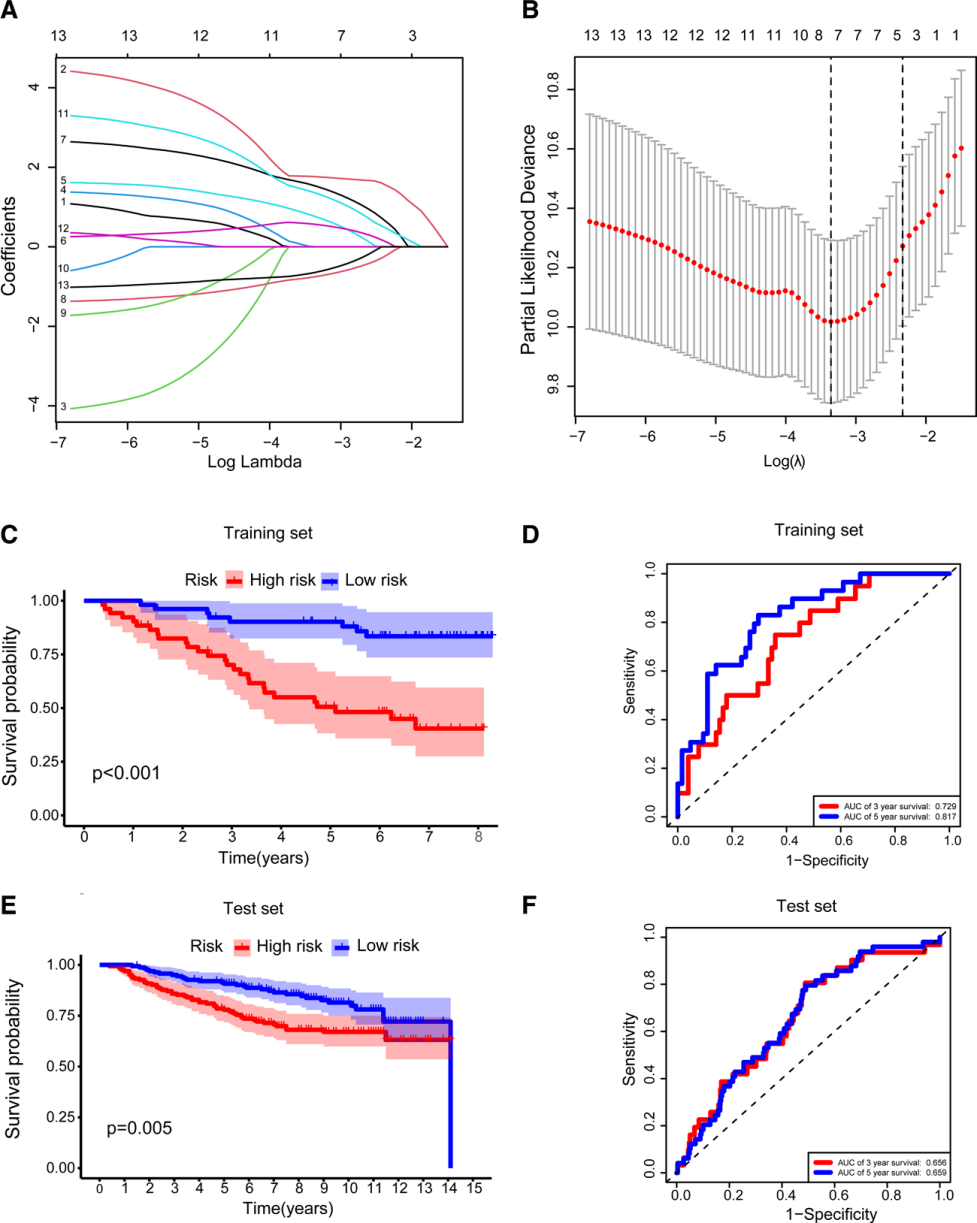
Construction and evaluation of prognostic URG signature for breast cancer patients. (A) LASSO coefficient profiles of differentially expressed URGs. (B) “Leave-one-out-cross-validation” for parameter selection in the LASSO model. (C, E) Kaplan–Meier curve of breast cancer patients according to the 4-URG signature in the training set and test set. (D, F) ROC curve of the 4-URG signature for predicting 3-year OS, and 5-year OS of BC patients in the training set and test set. LASSO = least absolute shrinkage and selection operator, OS = overall survival, ROC = receiver operating characteristic, URG = ubiquitination related gene.

A total 13 prognostic URGs were fitted into a LASSO Cox analysis to identify the optimal prognostic URGs in the training set. We recognized 5 URGs (CDC20, KLHL13, PCGF2, SOCS2 and UBE2S) using LASSO Cox analysis (Fig. 3A, B). Next, we applied multivariate Cox proportional hazards regression analysis to identify optimal URGs. We identified 4 URGs and constructed a prognostic signature by integrating the 4 URGs expression profiles and corresponding Cox regression coefficient. We calculated the risk score for each patient in the training group and ranked them into a high-risk group (n = 52) and a low-risk group (n = 52) according to the median of risk score. The Kaplan–Meier curve showed patients in high-risk group have significantly worse OS than patients in low-risk group (*P* < .001, Fig. 3C). The prognostic power of the 4-URG signature was evaluated by calculating the AUC. The results showed that the AUC of 4-URG signature for predicting 3-year survival, and 5-year survival of BC patients was 0.729, and 0.817, respectively, which indicated good performance (Fig. 3D). To verify the reliability of this 4-URG signature in BC, we applied this signature in the test set (GSE20685). We calculated the risk score for each patient in the test set and ranked them into a high-risk group (n = 164) and a low-risk group (n = 163). As presented in Figure 3E, patients in high-risk group have significantly worse OS than patients in low-risk group (*P* = .005). The AUC of the 4-URG signature for predicting 3-year survival, and 5-year survival in the test set was 0.656, and 0.659 respectively (Fig. 3F).

### 3.3. Independence of the 4-URG signature in BC

**Figure 4.**
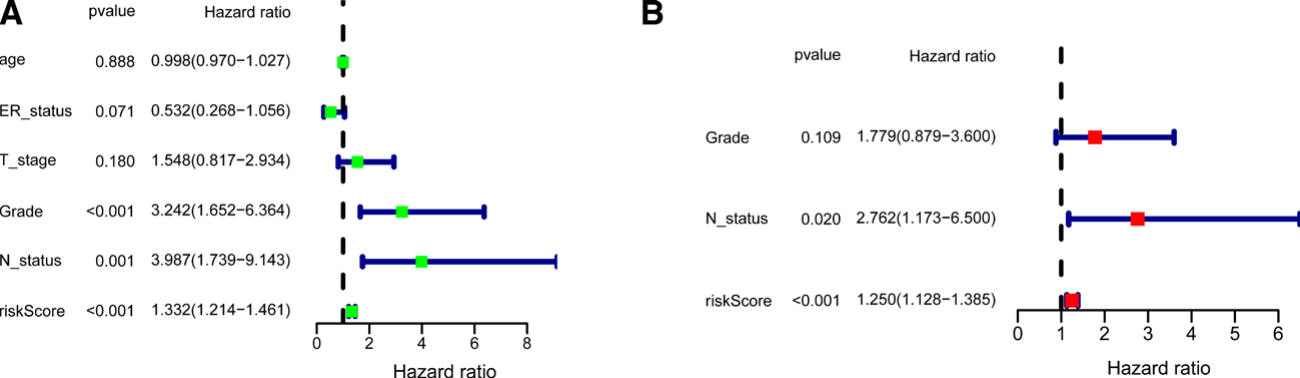
Forest plots of the 4-URG signature and clinicopathological factors for predicting prognosis of patients with breast cancer. (A) Univariate Cox regression analysis. (B) Multivariate Cox regression analysis. URG = ubiquitination related gene.

**Figure 5.**
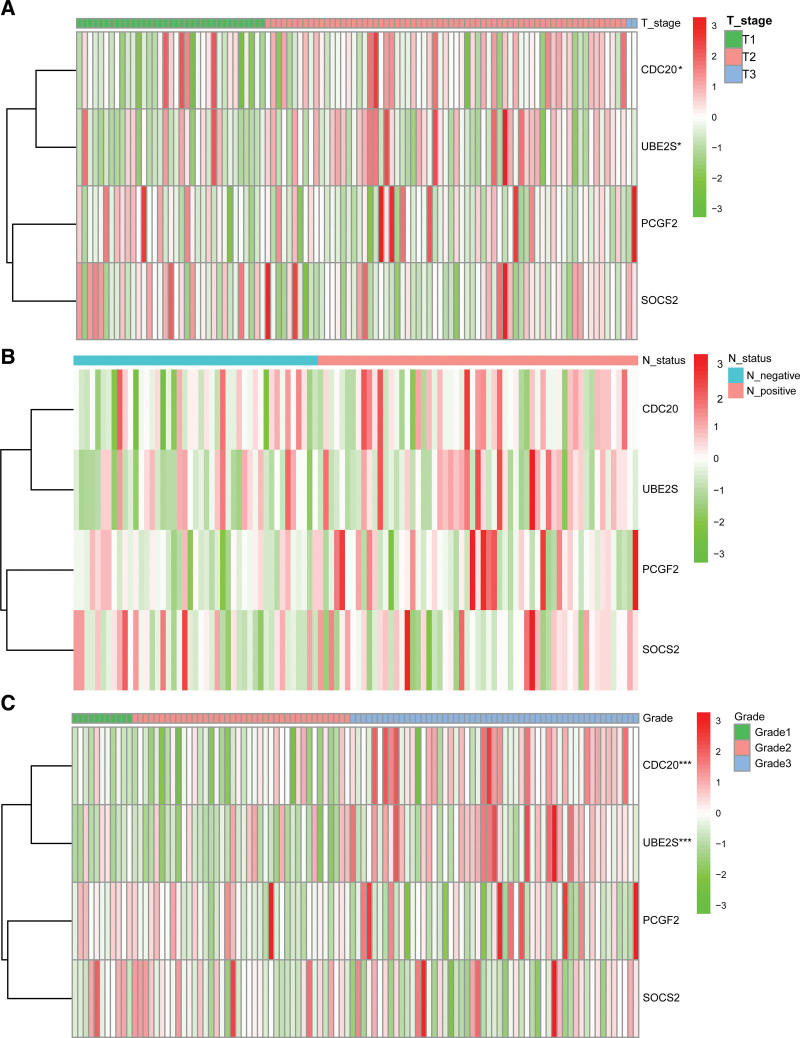
The correlation of 4 URGs with clinicopathological factors of breast cancer. Heatmap of the expression of 4 URGs with clinicopathological factors of breast cancer (A) T stage, (B) N stage, (C) different grade. URGs = ubiquitination related genes. *P < .05; ***P < .001.

In the training test, univariate Cox regression analysis suggested that the 4-URG signature based risk score, lymph node status, and grade were significantly associated with patients’ survival (Fig. 4A). We further preformed multivariate Cox regression analysis using these factors. The results revealed that the 4-URG signature and lymph node status were related with the OS of BC patients (Fig. 4B). Univariate and multivariate Cox regression analyses in the test set also indicated the 4-URG signature was an independent risk factors for predicting prognosis of BC patients (see Figure S1, Supplemental Digital Content 1, http://links.lww.com/MD/H329). As displayed in Figure 5, BC patients with grade 3 tend to have higher expression of CDC20 and UBE2S than patients with grade 2 and grade 1. Compared with T1 stage BC patients, T2 stage BC patients tend to have higher expression of CDC20 and UBE2S (Fig. 5A and Figure S2, Supplemental Digital Content 2, http://links.lww.com/MD/H330).

### 3.4. Gene set enrichment analysis between high-risk group and low-risk group patients

**Figure 6.**
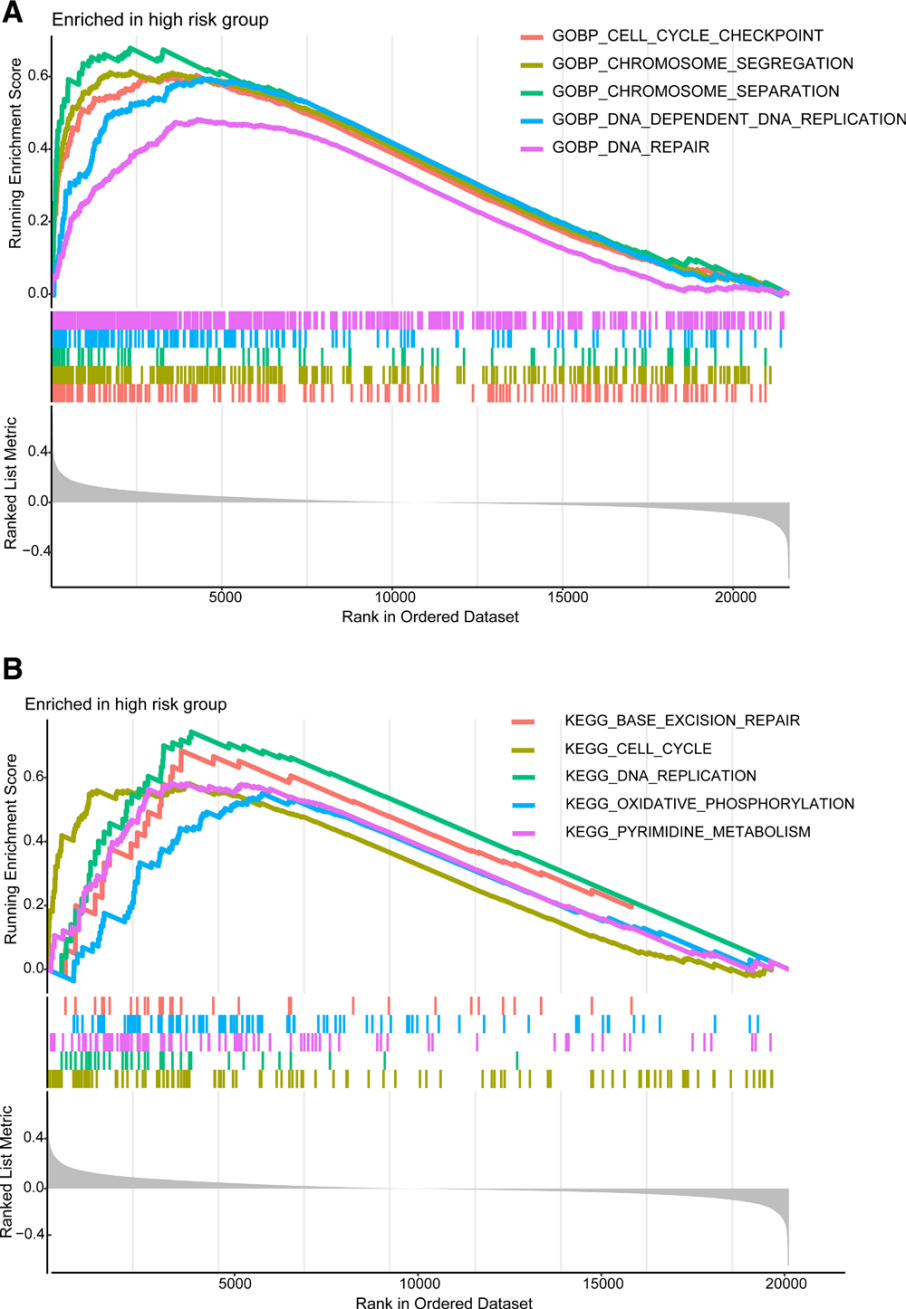
Gene set enrichment analysis in high-risk group. (A) Biological process enriched in high-risk group. (B) KEGG pathways enriched in high-risk group. KEGG = Kyoto Encyclopedia of Genes and Genomes.

To explore the difference of biological characteristics between high-risk and low-risk group patients, we performed GSEA analysis. The GSEA results were presented in Figure 6. Biological process of DNA repair, DNA replication, chromosome separation, and cell cycle checkpoint were enriched in high-risk group (Fig. 6A). As to Kyoto Encyclopedia of Genes and Genomes pathways, cell cycle, DNA replication, oxidative phosphorylation and base excision repair were the mainly enriched pathways in high-risk group (Fig. 6B).

## 4. Discussion

In the present study, we developed a 4-URG signature with good performance for patients with BC. According to the 4-URG signature, BC patients can be classified into a high-risk and low-risk group with significantly different OS. The 4-URG signature was validated in the test set (GSE20685). Univariate and multivariate Cox regression analyses showed that this signature was an independent risk factor for BC patients. GSEA analysis revealed that the 4-URG signature may related to the function of DNA replication, and cell cycle.

Conventional clinicopatholgic predictors such as age, gender, and TNM staging system are insufficient to predict the prognosis of breast patients due to molecules complexity and biological heterogeneity of BC. To provide a quantitative tool for predicting the survival rate of TNBC patients, we constructed a 4-URG signature based risk score. ROC curve suggested the signature is a stable and reliable predictor for OS of BC patients.

Of the 4 URGs, CDC20, PCGF2, and UBE2S are risk factors of BC, and SOCS2 is protective factor of BC. We further discussed the functions of these URGs. The factor CDC20, cell division cycle 20, acts as a regulatory protein interacting with several other proteins at multiple points in the cell cycle. The study by Song et al^[21]^ found that knockdown of CDC20 reduced triple-negative BC cell growth and migration. In addition, CDC20, as a substrate receptor of ubiquitin ligase Anaphase-Promoting Complex/Cyclosome, mediated degradation of SMAR1 and promoted cell migration and invasion in BC cell lines.^[22]^ The results of our study indicated the expression of CDC20 was higher in high stage and grade BC. Our study supports the evidence that CDC20 is an oncogene of BC and promotes tumor growth and metastasis. PCGF2, also named as MEL-18, encoded protein containing a ring finger motif. The role of PCGF2 in BC is controversial. Silva et al^[23]^ measured the expression PCGF2 and other polycomb group members in a series of 134 BC samples. They found PCGF2 expression correlated with the cell cycle regulators, but they did not analyze the prognostic role of PCGF2 in BC. Lee et al^[24–27]^ conducted a series of studies about the role of PCGF2 in BC. They found that PCGF2 prevent trastuzumab resistance in HER2 positive BC.^[26]^ Besides, they found that PCGF2 mediated Akt phosphorylation, and further promoted cyclin D1 expression and p27 phosphorylation in BC cell lines, through which BC cell growth was attenuated and G(1)-S phase transition was decelerated.^[27]^ In addition, they reported that PCGF2 inhibited epithelial-mesenchymal transition of BC cells, the number and self-renewal activity of BC stem cells.^[24,25]^ The study by Guo et al^[28]^ indicated that PCGF2 repressed Akt activity in BC cells. Guo et al^[29]^ concluded that PCGF2 was conversely correlated with the pathological classifications and served as a protective factor for BC. However, Mai et al^[30]^ found that AKT1 directly phosphorylated PCGF2 and promoted malignant behaviors in BC. Therefore, PCGF2 may have versatile roles in BC. In our study, we found that PCGF2 is upregulated in BC tissues and functions as a risk factor for BC. We speculate that PCGF2 may play a tumor suppressing role or act as an oncogene by affecting the ubiquitination levels of different substrate proteins. The functions of PCGF2 in BC require further study. UBE2S may regulate cell cycle by cooperating with CDC20, and Anaphase-Promoting Complex/Cyclosome to build K11-linked ubiquitin chains on substrates to target them for proteasomal degradation.^[31]^ However, the role of UBE2S in BC has not been fully characterized. Our study revealed that UBE2S was higher in grade3 than in grade1 and grade2. Moreover, UBE2S was negatively related to OS of BC. Taken together, UBE2S may act as an oncogene in BC and require further research. SOCS2 is a member of the suppressor of cytokine signaling family.^[32]^ SOCS2 was reported to inversely correlate with histopathological grade of BC and acted as a suppressor,^[33,34]^ which confirms the reliability of our results.

We conducted GSEA analysis to explore the mechanism of OS difference between high-risk and low-risk group. Cell cycle, DNA repair, and DNA replication related biological process and pathways were the mainly enriched items in high-risk group. DNA replication is one of the fundamental biological processes in which dysregulation can cause genome instability and contribute to cancer etiology.^[35]^ DNA damage repair processes are formed by cells to maintain genome stability. Defects in the DNA damage repair processes may cause cancer.^[36]^ Moreover, impaired DNA damage repair may affect the sensitivity of BC cells to certain drugs including chemotherapy drugs, targeted therapy drugs, and immunotherapy drugs.^[37]^ As is known, deregulation of the cell cycle is a hallmark of cancer that enables limitless cell division. Deregulation of the cell cycle is frequently observed in BC.^[38]^ Therapeutic targeting of the cell cycle for BC has emerged as a promising anticancer strategy.

Admittedly, our study has some limitations because it was based on the public database and data analysis only. The roles of these prognostic URGs deserve further in vitro and in vivo studies because of their strong relevance with prognosis of BC.

## 5. Conclusions

In conclusion, we identified a novel 4-URG signature with good performance for patients with BC. Moreover, GSEA analysis showed that the 4-URG signature may related to the function of DNA replication, DNA repair, and cell cycle. Our analysis suggests URGs play important roles in BC and were potential prognostic biomarkers.

## Author contributions

YZ and WL performed the research, analyzed and interpreted data, and drafted the manuscript. BC participated in data analysis. KZ participated in research design.

**Conceptualization:** Kankan Zhao.

**Data curation:** Yuan Zheng, Bo Chen.

**Formal analysis:** Yuan Zheng, Wenliang Lu.

**Supervision:** Kankan Zhao.

**Validation:** Wenliang Lu, Bo Chen.

**Visualization:** Yuan Zheng, Wenliang Lu.

**Writing – original draft:** Yuan Zheng.

**Writing – review & editing:** Kankan Zhao.

## Supplementary Material


