# Supplementary material for: Identification of a novel ubiquitination related gene signature for patients with breast cancer
Source: Medicine (Baltimore). 2022 Sep 16;101(37):e30598. doi: 10.1097/MD.0000000000030598 (PMC9478291; doi:10.1097/MD.0000000000030598)

Figure S1 Forest plots of the four-URG signature and clinicopathological factors for predicting prognosis of patients with breast cancer in test set (GSE20685). (A) Univariate Cox regression analysis. (B) Multivariate Cox regression analysis. URGs, ubiquitination related genes.

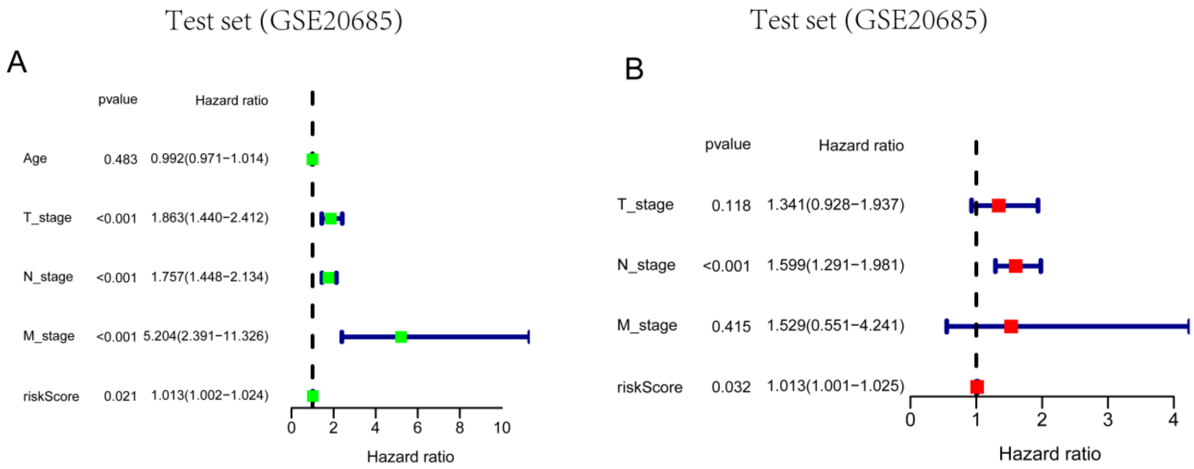

Supplement: Supplementary file 1 [file medi-101-e30598-s001.pdf]
