# Supplementary material for: Identification of a novel ubiquitination related gene signature for patients with breast cancer
Source: Medicine (Baltimore). 2022 Sep 16;101(37):e30598. doi: 10.1097/MD.0000000000030598 (PMC9478291; doi:10.1097/MD.0000000000030598)

Figure S2 The correlation of four URGs with T stage of breast cancer. Heatmap of the expression of four URGs with T stage of breast cancer in (A) TCGA. (B) GSE20685. URGs, ubiquitination related genes; * p < 0.05; ** p < 0.01; *** p < 0.001.

A

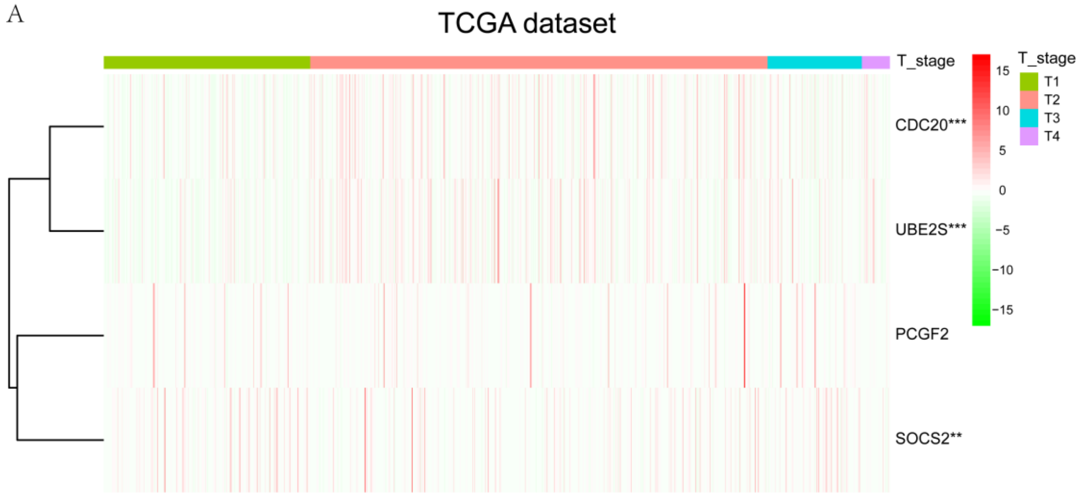

B

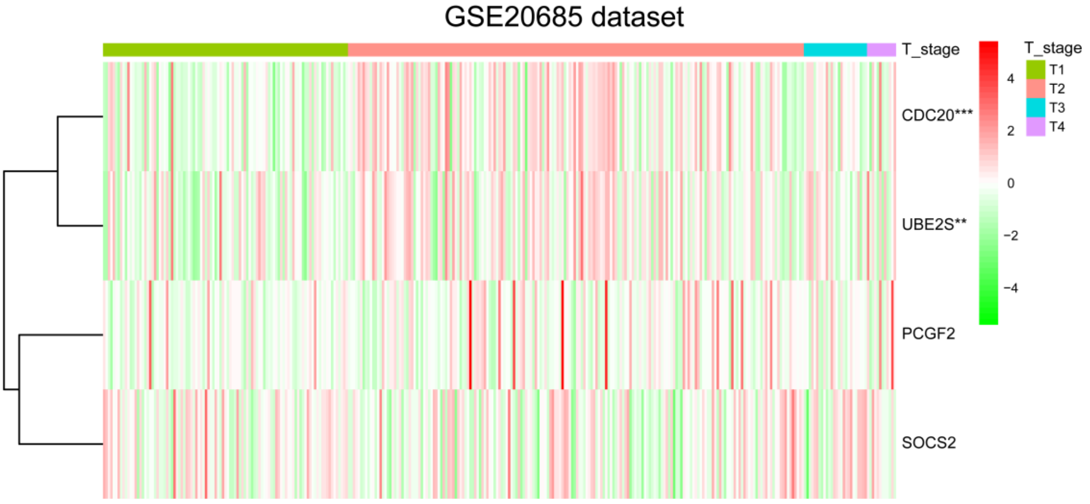

Supplement: Supplementary file 2 [file medi-101-e30598-s002.pdf]
